# Supplementary figures and images for: Homogeneity in the association of body mass index with type 2 diabetes across the UK Biobank: A Mendelian randomization study
Source: PLoS Med. 2019 Dec 10;16(12):e1002982. doi: 10.1371/journal.pmed.1002982 (PMC6903707; doi:10.1371/journal.pmed.1002982)

A. PRS additional quantiles

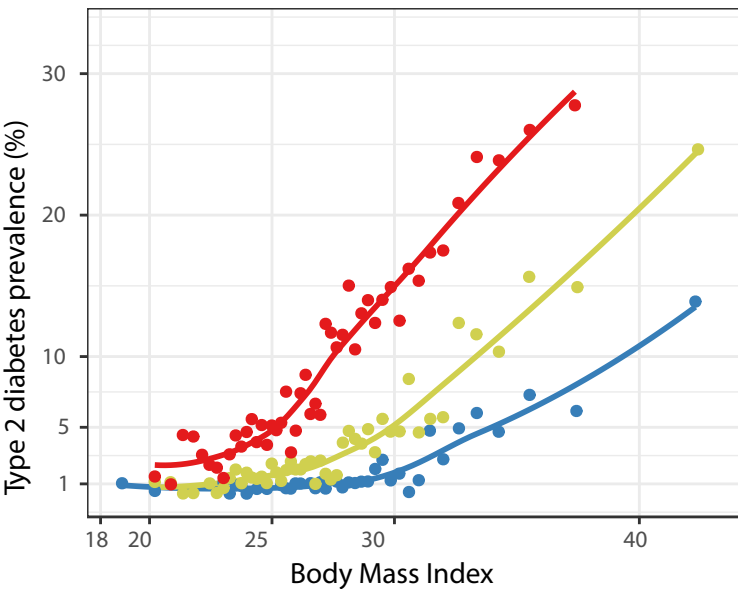

PRS quantile    0-5%    47.5-52.5%    95-100%

B. Sex-stratified T2D prevalence by BMI

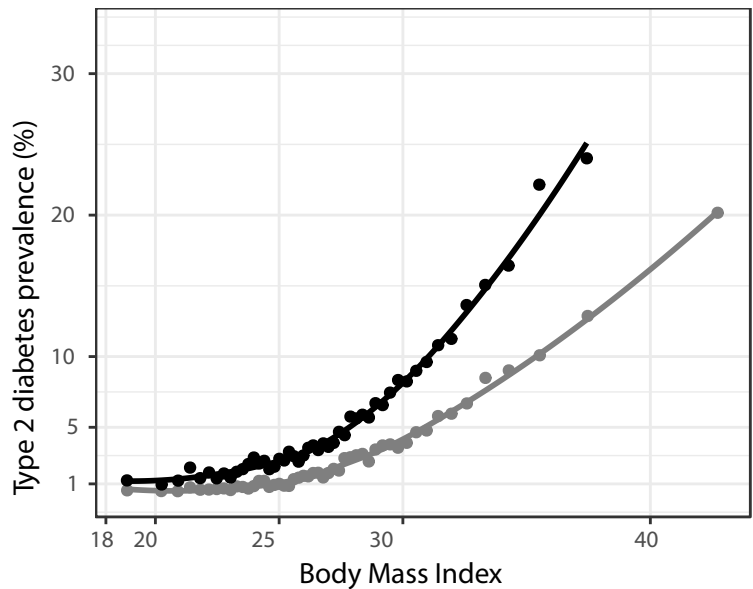

Sex    Female    Male

Supplement: S1 Fig — Within each category, 50 bins of equal numbers of individuals with consecutive BMI were selected, and the average BMI and percent of individuals diagnosed with type 2 diabetes are shown. Curves were fit using LOESS regression [33]. Individuals were stratified by (A) sex or (B) polygenic risk score quantile (0%–5%, 47.5%–52.5%, 95%–100%). (PDF) [file pmed.1002982.s001.pdf]

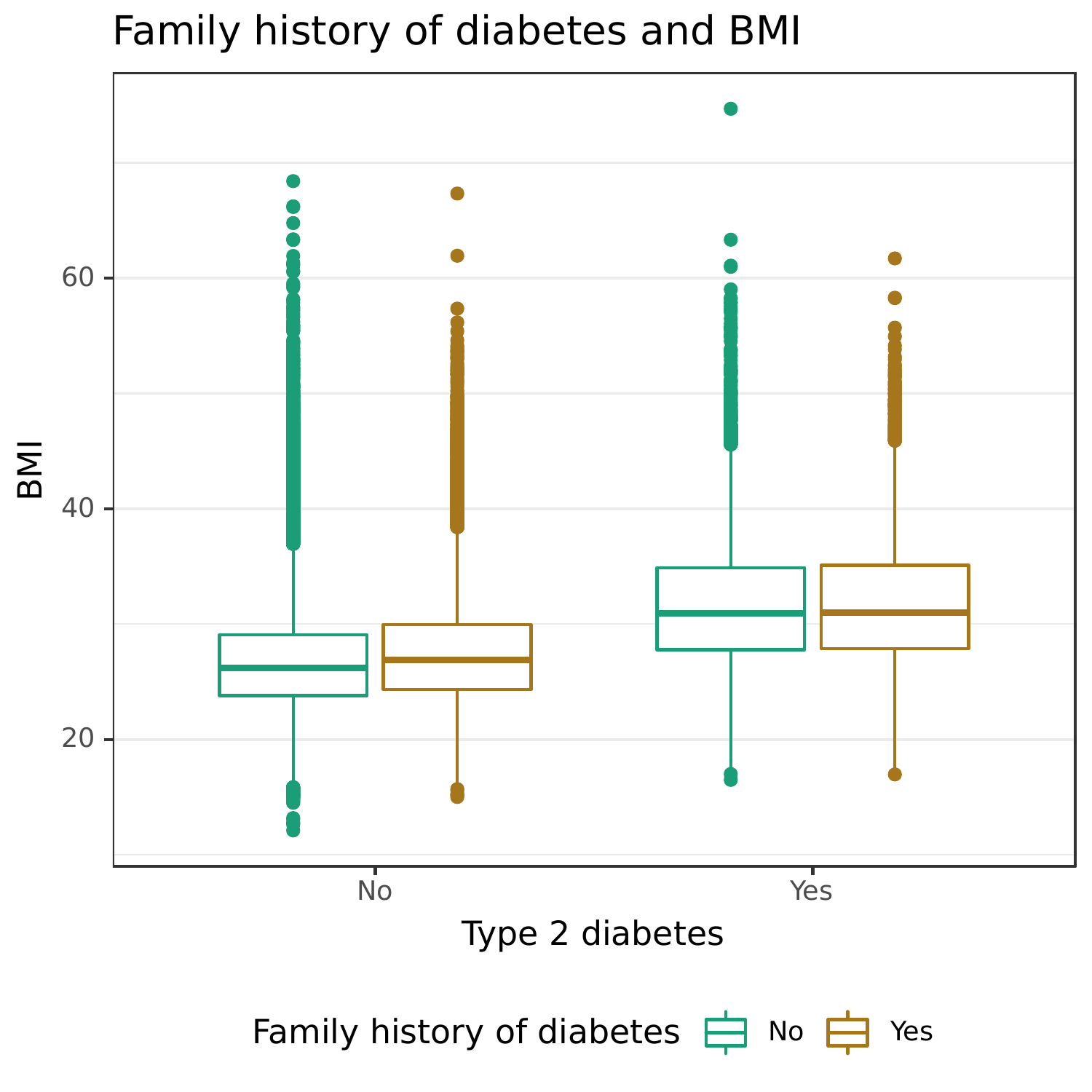

Supplement: S2 Fig — Individuals were stratified into controls (left) and type 2 diabetes cases (right) and further stratified by the presence (brown) or absence (green) of a family history of diabetes. There was a significant difference in mean BMI between individuals with a family history of diabetes in controls (0.8 kg/m2, t test p < 0.001) but not in cases (0.2 kg/m2, t test p = 0.1). (PNG) [file pmed.1002982.s002.png]

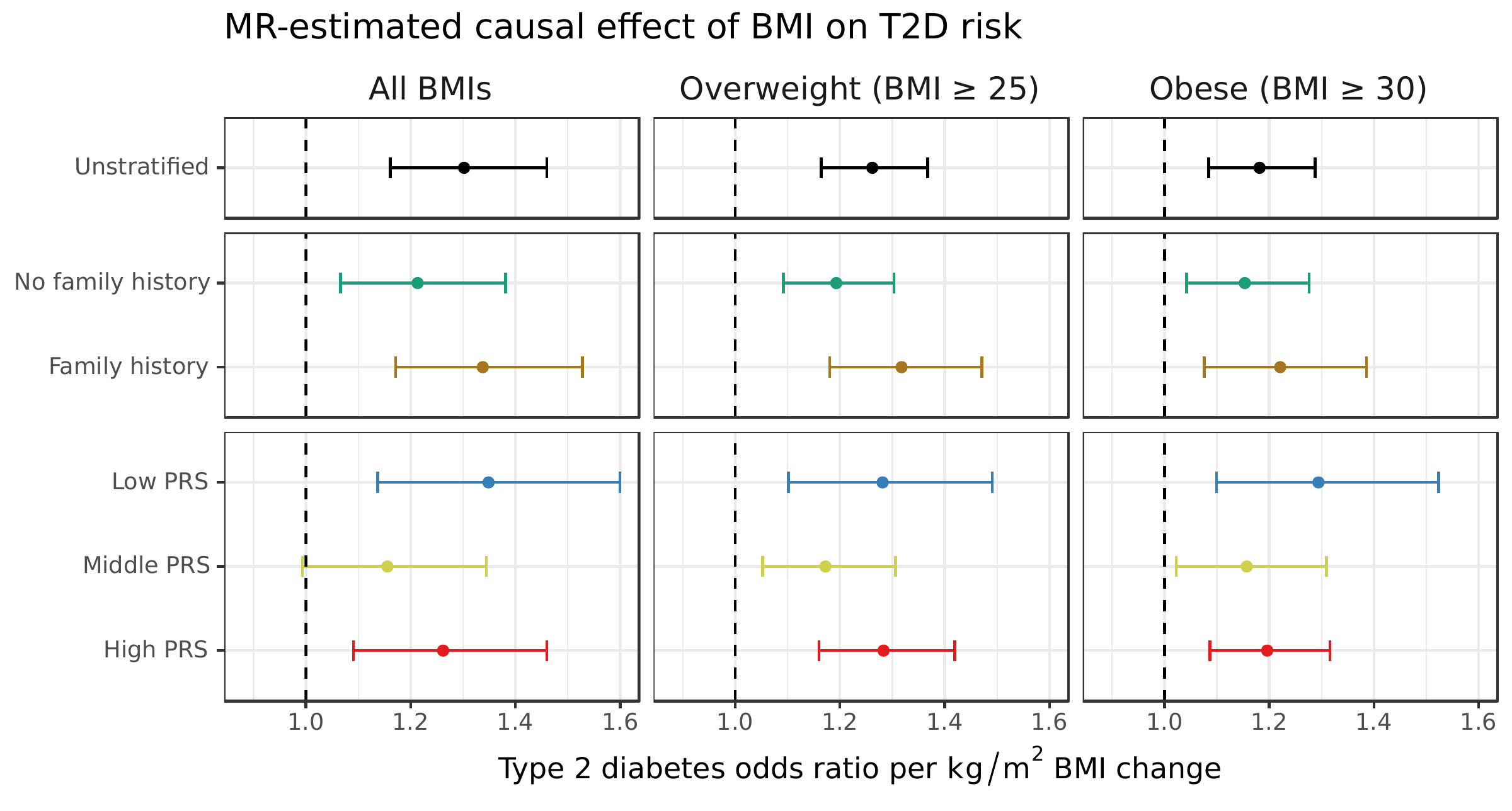

Supplement: S3 Fig — Error bars indicate 95% confidence intervals. (PNG) [file pmed.1002982.s003.png]

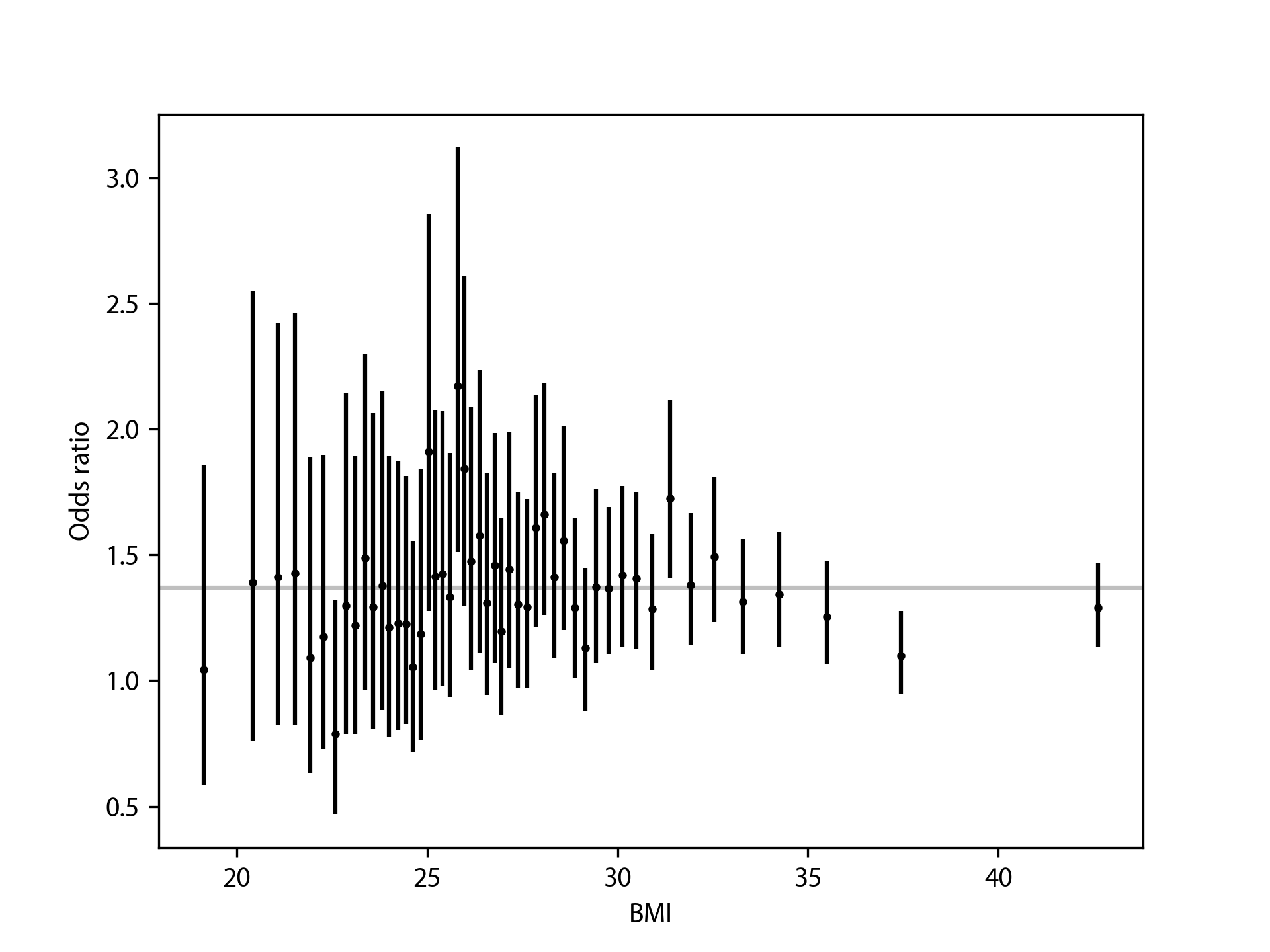

Supplement: S4 Fig — The average of the 50 odds ratios is shown as a gray line. We found no significant evidence of heterogeneity across the 50 quantiles (Cochran Q heterogeneity p = 0.6). However, the association of the allele score with BMI appeared to vary slightly across the 50 quantiles (Cochran Q heterogeneity p = 0.01, trend test p = 0.7), limiting the conclusions that can be drawn from this analysis. (PNG) [file pmed.1002982.s004.png]
