# Supplementary material for: Homogeneity in the association of body mass index with type 2 diabetes across the UK Biobank: A Mendelian randomization study
Source: PLoS Med. 2019 Dec 10;16(12):e1002982. doi: 10.1371/journal.pmed.1002982 (PMC6903707; doi:10.1371/journal.pmed.1002982)
Supplement: S1 Table — (DOC) [file pmed.1002982.s005.doc]

|  | **Non-overweight (BMI < 25)** | **Overweight**  **(25 ≤ BMI < 30)** | | **Obese**  **(BMI ≥ 30)** |
| --- | --- | --- | --- | --- |
| **Overall** | 0.6 | | **0.03** | |

| **No family history** | 0.1 | 0.8 | 0.9 | 0.1 | 0.5 |
| --- | --- | --- | --- | --- | --- |
| **Family history** |  | 0.1 |  | 0.09 |  |

| **Low PRS** | 0.2 | 0.6 | 0.6 | 0.6 | 0.4 |
| --- | --- | --- | --- | --- | --- |
| **Medium PRS** |  | 0.3 |  | 0.5 |  |
|  | 0.08 |  | 0.6 |  | 0.6 |
| **High PRS** |  | 0.1 |  | **0.02** |  |

| **Insulin only** | 0.8 | 1 |
| --- | --- | --- |
| **Metformin only** | 0.8 | **0.008** |

**Table S1: p-values between all adjacent groups in Table 2, calculated via a difference-of-odds-ratios test.** Each box spans a pair of groups from Table 2, and the number inside the box is the p-value corresponding to how significantly different the odds ratios of the two groups are from each other. Given a pair of odds ratios OR_1_ and OR_2_, corresponding to regression coefficients β_1_ and β_2_ with standard errors σ_1_ and σ_2_, the z-score corresponding to their difference is z_diff_ = (β_1_ - β_2_) / sqrt(σ_1_^2^ + σ_2_^2^). This z-score can be converted into a p-value p_diff_, which is what is shown in this table. Significant p-values are bolded.
