# Supplementary material for: Homogeneity in the association of body mass index with type 2 diabetes across the UK Biobank: A Mendelian randomization study
Source: PLoS Med. 2019 Dec 10;16(12):e1002982. doi: 10.1371/journal.pmed.1002982 (PMC6903707; doi:10.1371/journal.pmed.1002982)
Supplement: S2 Table — (DOC) [file pmed.1002982.s006.doc]

|  | **All BMIs** | **Low overweight**  **(BMI ≥ 25)** | | **High overweight**  **(BMI ≥ 27.5)** | | **Low obese**  **(BMI ≥ 30)** | | **High obese**  **(BMI ≥ 35)** |
| --- | --- | --- | --- | --- | --- | --- | --- | --- |
| **Prevalence** | 1.3% | 2.6% | | 5.2% | | 9.8% | | 21.1% |
| **Odds ratio** | 1.31  [1.11, 1.53] | 1.44  [1.31, 1.59] | | 1.32  [1.23, 1.43] | | 1.38  [1.29, 1.47] | | 1.13  [1.05, 1.21] |
| **p** | 0.3 | | 0.18 | | 0.44 | | **6 × 10^-5^** | |

**Table S2: Diabetes prevalence and odds ratio per kg/m^2^ change in BMI computed via MR, as in Table 2, but with more fine-grained stratifications for overweight and obese individuals.**  95% confidence intervals are indicated in square brackets; significant p-values (calculated via the same difference-of-odds-ratios test as in Table S1) are bolded.
