# Supplementary material for: Homogeneity in the association of body mass index with type 2 diabetes across the UK Biobank: A Mendelian randomization study
Source: PLoS Med. 2019 Dec 10;16(12):e1002982. doi: 10.1371/journal.pmed.1002982 (PMC6903707; doi:10.1371/journal.pmed.1002982)
Supplement: S3 Table — (DOC) [file pmed.1002982.s007.doc]

|  | **Non-overweight (BMI < 25)** | **Overweight**  **(25 ≤ BMI < 30)** | **Obese**  **(BMI ≥ 30)** |
| --- | --- | --- | --- |
| **Overall** | 1.05 [0.76, 1.44] | 1.36 [1.20, 1.53] | 1.18 [1.08, 1.29] |

| **No family history** | 1.07 [0.71, 1.63] | 1.23 [1.06, 1.42] | 1.14 [1.04, 1.25] |
| --- | --- | --- | --- |
| **Family history** | 0.93 [0.62, 1.39] | 1.41 [1.15, 1.72] | 1.22 [1.08, 1.39] |

| **Low PRS** | 1.06 [0.54, 2.10] | 1.27 [0.95, 1.69] | 1.29 [1.10, 1.52] |
| --- | --- | --- | --- |
| **Medium PRS** | 0.88 [0.51, 1.54] | 1.12 [0.88, 1.43] | 1.16 [1.02, 1.31] |
| **High PRS** | 0.91 [0.57, 1.44] | 1.52 [1.28, 1.79] | 1.20 [1.09, 1.32] |

| **Insulin only** | 1.08 [0.53, 2.21] | 1.49 [1.08, 2.06] | 1.14 [0.98, 1.33] |
| --- | --- | --- | --- |
| **Metformin only** | 0.96 [0.58, 1.60] | 1.41 [1.21, 1.66] | 1.21 [1.10, 1.34] |

**Table S3: The results of Table 2 using MR-Egger instead of inverse-variance-weighted MR.**
