# Supplementary material for: Homogeneity in the association of body mass index with type 2 diabetes across the UK Biobank: A Mendelian randomization study
Source: PLoS Med. 2019 Dec 10;16(12):e1002982. doi: 10.1371/journal.pmed.1002982 (PMC6903707; doi:10.1371/journal.pmed.1002982)
Supplement: S4 Table — (DOC) [file pmed.1002982.s008.doc]

|  | **Non-overweight (BMI < 25)** | **Overweight**  **(25 ≤ BMI < 30)** | **Obese**  **(BMI ≥ 30)** |
| --- | --- | --- | --- |
| **Overall** | 0.1 | 0.9 | 0.1 |

| **No family history** | 0.2 | 0.2 | **0.03** |
| --- | --- | --- | --- |
| **Family history** | 0.4 | 0.6 | 0.8 |

| **Low PRS** | 0.8 | 0.9 | 0.4 |
| --- | --- | --- | --- |
| **Medium PRS** | **0.02** | 0.09 | 0.05 |
| **High PRS** | 0.2 | 0.3 | 0.3 |

| **Insulin only** | 0.4 | 0.4 | **0.03** |
| --- | --- | --- | --- |
| **Metformin only** | 0.08 | 0.5 | 0.1 |

**Table S4: MR-Egger intercept test p-values.** Few p-values were significant (bolded), and then only barely, suggesting that horizontal pleiotropy does not substantially confound the analysis.
