# Supplementary material for: Homogeneity in the association of body mass index with type 2 diabetes across the UK Biobank: A Mendelian randomization study
Source: PLoS Med. 2019 Dec 10;16(12):e1002982. doi: 10.1371/journal.pmed.1002982 (PMC6903707; doi:10.1371/journal.pmed.1002982)
Supplement: Attachment — Submitted filename: Response to reviewers.pdf [file pmed.1002982.s009.pdf]

Requests from the editors:

Please avoid causal language as this study is not a trial – please remove the word 'effect' from the title and any other incidences in the manuscript.

Similar line 1 of the Methods and Findings section of the abstract (causal - Mendelian Randomization (MR) study of the causal effect size of BMI on diabetes odds in 287,399 unrelated individuals of white British ancestry in the UK Biobank) and causal appears multiple times in the main text.

We've updated the title to "Homogeneity in the Association of Body Mass Index with Type 2 Diabetes across the UK Biobank: A Mendelian Randomization Study". We've gotten rid of the word 'causal' as much as possible and have aimed to keep the word 'effect' only where it is clear that we are referring to the Mendelian Randomization effect size (in which case we make sure to use the term 'effect size' rather than 'effect').

Please provide p values in the abstract and elsewhere where any data is quantified with 95%Cis.

We've added p-values between adjacent groups for the MR results in Table 2 (as a separate table, Table S1) and in Table S2.

Please add a sentence as the final sentence of the Methods and Findings section of the abstract on the study's limitations.

We added the following sentence to the abstract: "A key limitation of this analysis is that the BMI-altering DNA sequence polymorphisms it studies represent cumulative predisposition to altered BMI over an individual's entire lifetime, and may consequently incorrectly estimate the risk-modification potential of interventions later in life". We'd be happy to expand on this sentence to include other limitations if you would like.

Please provide some summary demographic data to go into an abstract from the cohort you use in your study.

We've expanded the description of the cohort in the abstract: "287,399 unrelated individuals of white British ancestry in the UK Biobank, who were recruited from across the United Kingdom from 2006 to 2010 when they were between the ages of 40 and 69".

At this stage, we ask that you include a short, non-technical Author Summary of your research to make findings accessible to a wide audience that includes both scientists and non-scientists. The Author Summary should immediately follow the Abstract in your revised manuscript. This text is subject to editorial change and should be distinct from the scientific abstract. Please see

our author guidelines for more information:
https://journals.plos.org/plosmedicine/s/revising-your-manuscript#loc-author-summary

We've added an author summary following the abstract.

Academic Editor comments:

My only query/concern is on the 57 variants that were used to define the instrument. This is based on a 2015 GWAS, and variants curated from a previous MR analysis from a different group. It's good to have consistency between publications, but there have been many more variants associated now.

One reviewer commented:

"Is the opinion of this reviewer that the instrument should be updated accordingly", and another also raised concerns.

I would be interested in the statistical reviewer's opinion. I don't feel strongly either way, but it should probably be discussed in the paper.

Thanks for this comment, we agree this needs to be clarified. Reviewer 1 mentioned the possibility of using Yengo et al. 2018, which has over 530 independent BMI-associated SNPs. However, using Yengo would lead to circularity since it's a meta-analysis of Locke et al. (the GWAS that found the 69 SNPs) and UK Biobank, and MR instruments should be chosen from an external GWAS (as in, one that does not include UK Biobank data) to avoid winner's curse. We added a discussion of this point to the "Mendelian Randomization" section of the methods (bottom of page 6).

Reviewer #1:

In this manuscript, Michael Wainberg and colleagues investigated the effect of body mass index (BMI) on type 2 diabetes (T2D) using multi-stratum Mendelian randomisation (MR) analysis in the UK Biobank. Authors showed that genetically driven BMI reduction was consistently associated with reduced T2D odds across BMI, family history, and genetic risk categories. Findings suggest that all individuals can substantially reduce their T2D risk through weight loss.

The topic, as pointed out by the authors, is of high interest given evidence from large high-quality randomized clinical trials showing that lifestyle interventions can prevent or delay the progression to T2D by ~50% among high-risk individuals. The present study attempted to extend these observations in low-risk individuals using contemporary methodological approaches. The corroboration that body weight reduction strategies may work even among individuals at low T2D risk could offer a rational framework for practice and policy by supporting

dietary or lifestyle interventions for T2D to be deployed across all gradients of the population. The manuscript is clear, concise, and very-well written.

This paper has the following strengths:

1. Focus on an interesting and relevant hypothesis
2. Well-powered study with appropriate genetic and outcome information
3. Robust statistical approach
4. Potential to inform practice and policy
5. Authors are top scientist in the field of obesity and T2D genetics

I would like to make the following suggestions in order to help improve the overall quality of the manuscript and strengthen the impact of main findings.

BMI is instrumented by the use of 57 BMI increasing-risk variants that passed the quality control threshold defined by the authors. Weights were based on Locke at al., data. There are now more than 530 independent loci associated with BMI (Yengo E, et al. Hum Mol Genet 2018) and some loci contain more than one causal SNP. Is the opinion of this reviewer that the instrument should be updated accordingly, otherwise they are building a genetic instrument based on a subset of variants that can be difficult to interpret. A potential solution to avoid the inclusion of poor quality variants is to identify proxies for loci in which the lead variant did not pass the quality control.

Thanks for this comment, we agree this needs to be clarified. Using Yengo would lead to circularity since it's a meta-analysis of Locke et al. (the GWAS that found the 69 SNPs that we filtered down to 57 after missingness and Hardy-Weinberg filters) and UK Biobank, and MR instruments should be chosen from an external GWAS (as in, one that does not include UK Biobank data) to avoid winner's curse. We added a discussion of this point to the "Mendelian Randomization" section of the methods (bottom of page 6).

The relevance of these findings on T2D call for the need to investigate hard outcomes such as coronary artery disease.

We agree that this is a vital question, and worthy of a separate investigation, but we believe it to be beyond the scope of the present paper. Indeed, though not specifically in a diabetes context, Khera et al. 2016 (PMID: 27959714) have investigated the interplay of genetic risk and lifestyle choices on 10-year coronary event rates in three prospective cohorts.

Reviewer #2:

The paper by Rivas and colleagues explores the causal effect of BMI reduction on risk of diabetes across the spectrum of diabetes risk using MR. This is an important question and I

found the manuscript to be well-balanced in its methodological approach and discussion. I do have some questions and suggestions for the authors:

1. The authors constructed an allele score from the weighted sum of 57 alleles using GIANT results (Locke et al.). However, the authors also state "using correlation with BMI in UK Biobank to determine effect directions". Does this mean the direction of effect of (each of) the 57 polymorphisms determined in the UK Biobank for calculation of the allele score? This will certainly inflate the predictiveness of the instrument and bias results. Can the authors explain, and if this is not already the case, use the same direction of effect as in Locke et al.?

We apologize for the confusion here, we have removed this statement. For all instruments, the effect directions are the same between Locke et al. and Biobank, so strand-flipping is handled correctly by using the UK Biobank effect directions.

2. Why did the authors choose MR-Egger? Can the authors provide results using alternative method (IVW-MR and weighted median, for example)? Was horizontal pleiotropy a concern? If not, other methods are likely to be better powered.

We have switched to IVW in the main text and now report MR-Egger results in the supplement (Table S3), along with p-values for the MR-Egger intercept test, which are generally non-significant (Table S4). Note that weighted median, like Egger, also sacrifices power for horizontal pleiotropy correction.

3. A key assumption of the Staley and Burgess method (and all methods looking for non-linear relationships using MR in general) is that the effect of the IV on the exposure is linear and constant for all individuals across the entire of the exposure distribution. Have the authors checked this is the case?

We now report the trend test and Cochran Q test P-values for the associations of the IV with BMI across strata: Cochran Q heterogeneity $p = 0.01$, trend test $p = 0.7$. We now state in the caption to Figure S4 (bottom of page 24) that this "limit[s] the conclusions that can be drawn from this analysis".

4. It is unclear to me when and how the BMI genome-wide association study the authors have performed in each subset was used in results. If I understand correctly, 57 SNPs from the Locke paper were used as instruments, either with MR-Egger (and results of the diabetes GWAS in each subset) or as a weighted allele score for analysis of nonlinear relationships in 100 BMI quantiles. Can the authors clarify?

Yes, that's correct. For the stratified analysis, we took the 57 SNPs from Locke and calculated their BMI and type 2 diabetes effect sizes within each subset, then ran MR.

5. I suggest adding a column for non-overweight individuals (BMI<25) in Table 1. This would provide an estimate of baseline risk for individuals with a healthy BMI, as compared to "all BMI", which includes overweight and obese individuals.

We agree and have switched to non-overlapping categories (BMI < 25, BMI 25-30, and BMI > 30).

6. The authors have used a log scale for the y-axis of Figure 1. I wonder if this might mask the rather spectacular effect of BMI on diabetes risk. If felt appropriate, I would suggest using a linear scale.

We’ve switched to a linear scale for this figure.

7. The authors state (page 12) that "However, individuals with a family history of diabetes were predicted to have reduced risk-modification ability through BMI compared to individuals without." This is not readily apparent to me from Table 2. In fact, ORs were consistently larger for individuals with a family history as compared to without. Also, is this difference significant?

We apologize for the inclusion of that sentence and have taken it out. In the new analysis, the difference is not significant within any stratum ($p = 0.1$ for non-overweight, $p = 0.9$ for overweight, $p = 0.5$ for obese, see Table S2).

8. Figure 2 and Table 2 provide the same information. Would it be possible to combine?

It’s true that Figure 2 isn’t really necessary to have in the main text since all the information in it is also in Table 2. As a result, we’ve moved Figure 2 to the supplement as Figure S3.

9. I find it difficult to interpret some of the comparisons for predicted weight loss because the "overweight" category includes both overweight and obese individuals. It would be more intuitive to restrict the overweight category to overweight participants (i.e. BMI from 25 to 29.9). To some extent, it is not surprising the two categories have similar risk reduction as they are largely overlapping as currently defined.

We agree and have switched to non-overlapping categories (BMI < 25, BMI 25-30, and BMI > 30).

10. The labeling of Figure S2 does not match the figure legend.

We’ve removed the title “Family history of diabetes and BMI” from Figure S2.

11. Use of 100 BMI quantiles might obscure any non-linearity because of the large imprecision of estimates in each quantile (figure S3). Can the authors repeat using fewer quantiles?

We've switched to using 50 quantiles instead of 100.

Reviewer #3:

This manuscript uses data from the UK Biobank to examine the causal impact of body mass (BMI)/obesity on type 2 diabetes. They look at prevalence of type 2 diabetes stratified by BMI categories, diabetes family history, and diabetes risk scores. Subsequently, they used Mendelian Randomization across BMI categories to test the reduced T2D risk per kg/m^2 reduction in BMI. Despite increases in T2D prevalence across BMI categories, predicted risk reduction per unit decrease in BMI is roughly comparable ~20-30% decrease across categories. These results are all broadly consistent with previous estimates of the impact of BMI on T2D risk and strongly suggest that weight loss is an effective intervention for diabetes prevention across all weight and genetic/environmental risk categories. The example showing risk reduction for a sample person in terms of weight reduction is useful.

The paper is clearly written with a straightforward hypothesis. It provides clear examples of why this information is useful and indicates the similar utility of weight loss in T2D prevention for all strata. I have only a few questions/comments.

Comments:

1) My main question regarding the presentation of this analysis is why the results are presented with overlapping BMI categories? I can understand presenting the full distribution results (all BMIs), but it seems the analyses would be more logical if presenting discrete categories (such as typical BMI breakpoints <25, 25-30, >30) that are distinct from each other as opposed to categories that are nested within each other (>25, and >30).

We agree and have switched to non-overlapping categories (BMI < 25, BMI 25-30, and BMI > 30).

2) Since many more than 69 SNPs have been associated with BMI, I assume the 69 SNPs used (subsequently pruned to 57) were chosen due to significant association in European-descent samples, correct? Some comment about this specific selection is warranted.

Thanks for this comment, we agree this needs to be clarified. Reviewer 1 also mentioned this point and brought up the possibility of using Yengo et al. 2018, which has over 530 independent BMI-associated SNPs. However, using Yengo would lead to circularity since it's a meta-analysis of Locke et al. (the GWAS that found the 69 SNPs) and UK Biobank, and MR instruments should be chosen from an external GWAS (as in, one that does not include UK Biobank data) to avoid winner's curse. We added a discussion of this point to the "Mendelian Randomization" section of the methods (bottom of page 6).

3) In so far as a noticeable signal of differences exists in these data, it is potentially between those with a family history and those without. Family history encompasses both a shared genetic component and a shared environmental component. Can we use these data to tease apart the two aspects as to which is contributing to the slightly declined value of weight loss in preventing T2D with a family history of T2D? It looks, in comparing odds ratios to T2D PRS to be rather complex.

We apologize for the inclusion of the sentence “However, individuals with a family history of diabetes were predicted to have reduced risk-modification ability through BMI compared to individuals without”. The more accurate conclusion is that “Crucially, neither family history nor genetic risk fundamentally alter the strength of [the] association”, as we mention in the discussion (bottom of page 14). We’d be cautious about interpreting the difference due to family history as genetic vs environmental when there may not even be a real difference in the first place.

4) I am confused by Figure S3. I assume that the lines extending from the point estimates are confidence intervals of some sort (not indicated). Can you explain why the confidence intervals are largest in the center of the BMI distribution where the bulk of the data lies and smaller at the tails? This seems counterintuitive.

This could be because, even though there are the same number of individuals in each bin, the bin sizes are larger at the tails (in terms of the number of units of BMI they span) and therefore have more phenotypic variance within them. In general, greater phenotypic variance makes it easier to tell which variants are genuinely associated with the phenotype, i.e. reduces their GWAS standard errors, which in turn shrinks the error bars on the MR estimate.

Reviewer #4:

I confine my remarks to statistical aspects of this paper. These were very well done and I have no problems recommending publication.

We thank the reviewer for this comment.
