# Supplementary material for: Homogeneity in the association of body mass index with type 2 diabetes across the UK Biobank: A Mendelian randomization study
Source: PLoS Med. 2019 Dec 10;16(12):e1002982. doi: 10.1371/journal.pmed.1002982 (PMC6903707; doi:10.1371/journal.pmed.1002982)
Supplement: Attachment — Submitted filename: Response to editors.pdf [file pmed.1002982.s010.pdf]

FIGURES and GRAPHICS:

1. Please remove embedded figures from your manuscript file and ensure the most up to date versions of your figures are uploaded to our submission system as separate TIF or EPS files. Please do not remove the figure legends and captions.

We’ve removed the main-text figure.

2. Please ensure that all main figure files are cited in ascending numerical order in the main text of the article.

We’ve confirmed this.

3. Please ensure that all main figures are referenced in the manuscript as Fig 1, Fig 2, etc. (including capitalization), rather than Figure 1, Figure 2, etc. Please note, however, that the file names themselves must not include the space, i.e. Fig1.tif, Fig2.tif, etc.

We’ve changed “Figure” to “Fig” throughout.

4. Please ensure that all main figure legends are placed after the paragraph containing their first in-text citation.

We’ve moved the figure legend to the right spot.

TABLES and BOXES:

1. Please ensure that all main tables in their entirety (including titles and footnotes), are placed after the paragraph containing their first in-text citation.

We’ve moved the tables to the right spots.

2. Tables 1, 2, 3, 4 and 5 use returns to control text wrapping. This is indicated by a [¶ or ↵] symbol when "Show Paragraph Marks" is turned on in Word. Please either split those cells into separate cells to achieve alignment or remove the returns.

We’ve removed the returns.

METADATA:

1. Please accurately complete the 'Competing Interests' section on our submission form, including any COIs declared by your co-authors. If you have no competing interests to declare, please add "The authors have declared that no competing interests exist". Otherwise please

declare all competing interests beginning with the statement "I have read the journal's policy and the authors of this manuscript have the following competing interests:"

We've completed this section.

2. Your co-author, Dr. McCarthy, has declared the following competing interest in relation to this manuscript. Please include it in the main competing interest statement in our system, sticking as closely

as possible to Dr. McCarthy's wording, using only their initials. "McCarthy declared: MMcC is a Wellcome Investigator and an NIHR Senior Investigator. The views expressed in this article are those of the author(s) and not necessarily those of the NHS, the NIHR, or the Department of Health. He has served on advisory panels for Pfizer, NovoNordisk, Zoe Global; received honoraria from Merck, Pfizer, NovoNordisk and Eli Lilly; has stock options in Zoe Global; and has received research funding from Abbvie, Astra Zeneca, Boehringer Ingelheim, Eli Lilly, Janssen, Merck, NovoNordisk, Pfizer, Roche, Sanofi Aventis, Servier, Takeda. As of June 2019, MMcC is an employee of Genentech and a holder of stock in Roche."

Your co-author, Dr. Sinnott-Armstrong, has declared the following competing interest in relation to this manuscript. Please include it in the main competing interest statement in our system, sticking as closely as possible to Dr. Sinnott-Armstrong's wording, using only their initials. "Sinnott-Armstrong declared: I am funded by the Department of Defense through the National Defense Science and Engineering Grant and by a Stanford Graduate Fellowship."

We've included the competing interests for Mark McCarthy in the submission system. We included Nasa Sinnott-Armstrong's fellowship funding in the funding section.

3. Please ensure only the authors' initials are included in the competing interest statement, not their full names or titles.

We've ensured this.

4. In accordance with PLOS' data policy, please ensure that your Data Availability Statement in the submission form clearly identifies how readers can access your data. Data cannot be available on request, or only accessible by contacting one of the authors. Note that your Data Availability Statement will be typeset as it is written, so please ensure it is in complete sentences and appears as you would like it to in the published version.

For details on our data policy, please see http://journals.plos.org/plosmedicine/s/data-availability

We've confirmed that this statement is present in the submission form.

ARTICLE BODY:

1. Please indicate the corresponding author in the byline by placing an asterisk (*) after their affiliation number.

We’ve done this.

2. Please include the corresponding author's email address on the title page of your manuscript, indicated by an asterisk (*). Only an asterisk and the email address itself are required.

We’ve ensured the corresponding authors’ email addresses are included on the title page.

3. Please remove author degree titles from the byline of your article file.

We’ve taken them out.

4. The names for your co-authors Mark I. McCarthy and Manuel A. Rivas do not match across the article file and the submission form. Please ensure that all author names match verbatim, including the use of a middle name/initials and any special characters (accents, umlauts, etc.), as correct spelling and formatting of author names is vital for accurate indexing in PubMed.

We’ve added their middle initials to the submission form.

5. Please remove the funding information from your manuscript file. The published version is pulled automatically from the “Financial Disclosure” field in the submission form and must not be duplicated. Please therefore take this opportunity to check that the information in this field is correct and completed in full.

We’ve taken it out and confirmed that the version in the submission form is correct and complete.

6. Please remove the Competing Interests section from your manuscript file. The published version is pulled automatically from the "Competing Interests" field in the submission form and must not be duplicated. Please therefore take this opportunity to check that the information in this field is correct and completed in full.

We’ve taken it out and confirmed that the version in the submission form is correct and complete.

7. Please remove your Author Contributions section from your manuscript file as this information is automatically pulled from the authors section of our submission form and must not be duplicated. Please therefore take this opportunity to check that the information in this field is correct and completed in full.

We’ve taken it out and confirmed that the version in the submission form is correct and complete.

8. Please remove the data availability information from your manuscript file as this is automatically pulled from the "Data Availability" field in the submission form and must not be duplicated. Please therefore take this opportunity to check that the information in this field is correct and completed in full.

We’ve confirmed that we do not currently have a data availability section in the manuscript and that the version in the submission form is correct and complete.

ACKNOWLEDGMENTS:

1. Please remove the funding information from the Acknowledgments section of your manuscript file. The published version is pulled automatically from the “Financial Disclosure” field in our submission form and must not be duplicated. Please therefore take this opportunity to check that the information in this field is correct and completed in full.

We’ve removed the funding information from the Acknowledgments.

2. Please remove the competing interest information from the Acknowledgements section of your manuscript file. The published version is pulled automatically from the “Competing Interests” field in our submission form and must not be duplicated. Please therefore take this opportunity to check that the information in that field is correct and completed in full.

We’ve removed the competing interest information from the Acknowledgments.

3. According to PLOS policy, we ask that you please remove any mention of the reviewers or editors from the Acknowledgments section of your manuscript.

We’ve ensured there are no mentions of them.

4. Please move the disclaimer to the end of the Acknowledgments section of your manuscript, with a line break between the main text of the acknowledgments and the disclaimer statement.

We’ve removed the disclaimers.

SUPPORTING INFORMATION:

1. Please ensure your supporting information files are individually uploaded to the File Inventory as file type 'Supporting Information' and are not present in the manuscript file.

We’ve ensured this.

2. Please upload your supporting information as individual files, and use the following naming format: S1 Text; S1 Fig; S1 Table, S1 Data, etc. Each file type should start as S1 and increase numerically: S1 Text, S2 Text, etc. Note that all relevant in-text citations and captions will need to be updated accordingly.

We’ve uploaded these files.

3. Please ensure that all Supporting Information files use the following format verbatim throughout the manuscript (including legends and all in-text citations): S1 Fig, S1 Table, S1 Text, S1 Data, etc. Each file type should start at 1 and increase numerically. Please note, however, that the file names themselves must use an underscore rather than a space, i.e. S1_Fig.tif, S1_Table.xlsx, S1_Text.doc, S1_Data.xlsx, S1_PRISMA_Checklist.docx, etc.

We’ve ensured this.

4. Please ensure that each supporting information file has a legend at the end of the manuscript file, after the Reference list.

We’ve ensured this.

5. Please note that your submission cannot contain any references to generic "supporting information" files and instead must refer to specific files using our naming convention (S1 Fig, S1 Table, S1 Text, S1 Data, SI PRISMA Checklist, etc.)

We’ve ensured this.
